# Prevalence and pattern of total and partial anomalous pulmonary venous connection in Indian patients with congenital heart disease

**DOI:** 10.1186/1532-429X-17-S1-P222

**Published:** 2015-02-03

**Authors:** Vimal Raj, Varun Bhat, Vinay Belval, Karthik GA, Venkatraman Bhat

**Affiliations:** Imaging, Narayana Hrudayalaya Hospitals, Bangalore, India

## Background

Total Anomalous Pulmonary Venous Connection (TAPVC) is a rare form of congenital heart disease (CHD) in which all pulmonary veins connect to the systemic veins, right atrium or the coronary sinus. Echocardiography is a good screening modality in picking up majority of these cases. Cross sectional imaging is often utilized to establish the anatomy and confirm the diagnoses. In this study we looked at the prevalence and pattern of TAPVC and Partial Anomalous Pulmonary Venous Connection (PAPVC) in Indian patients with CHD.

## Methods

Retrospective analysis of all Multi Detector Computed Tomography (MDCT) and Cardiac Magnetic Resonance (CMR) examinations performed in our center over 8-year period (2006-2013). Patients with Anomalous Pulmonary Venous Connection (APVC) were classified into TAPVC and PAPVC. Patients with TAPVC were sub-classified into supra-cardiac, cardiac, infra-cardiac and mixed type based on the site of drainage.

## Results

A total of 3983 patients with CHD had cross sectional imaging during this period. Of these, 411 (10.3%) patients had APVC, 232 (6%) had TAPVC and 172 (4.3%) had PAPVC. The most common type of TAPVC was supra-cardiac in 106 (46%), followed by cardiac in 55 (24%) and mixed type of TAPVC in 51 (22%) patients. Infra-cardiac TAPVC was present in 20 (8%) cases. Cross sectional imaging was able to recognise all pulmonary veins accurately in these patients. It was also particularly useful in picking up cases with obstruction that required urgent surgical management.

## Conclusions

Anomalous pulmonary venous connection is a common form of CHD in Indian patients. Cross sectional imaging plays an important role in diagnosis and establishing the anatomy in these patients.

